# Effect of a workshop in rational pharmacotherapy for interns during family medicine clerkship in Samsun- Turkey

**Published:** 2014

**Authors:** Mustafa Fevzi Dikici, Fusun Yaris, Fusun Artiran Igde, Fulya Yarar, Oznur Altuntas, Aysenur Alper Gurz

**Affiliations:** 1 Mustafa Fevzi Dikici, MD, Associate Professor, Department of Family Medicine, Ondokuzmayis University School of Medicine, Samsun, Turkey.; 2 Fusun Yaris, MD, PhD, Professor, Department of Family Medicine, Ondokuzmayis University School of Medicine, Samsun, Turkey.; 3 Fusun Artiran Igde, MD, Associate Professor, Department of Family Medicine, Ondokuzmayis University School of Medicine, Samsun, Turkey.; 4 Fulya Yarar, MD, Department of Family Medicine, Ondokuzmayis University School of Medicine, Samsun, Turkey.; 5 Oznur Altuntas, MD, Department of Family Medicine, Ondokuzmayis University School of Medicine, Samsun, Turkey.; 6 Aysenur Alper Gurz, MD, Department of Family Medicine, Ondokuzmayis University School of Medicine, Samsun, Turkey.

**Keywords:** Rational drug therapy, Family medicine, Undergraduate medical education

## Abstract

***Objective:*** We aimed to investigate the effect of rational pharmacotherapy workshop for interns on the rationality, cost and number of drugs prescribed.

***Methods:*** The participants were asked to prescribe a medication for acute noninflammatory osteoarthritis (ANOA), acute bacterial rhinosinusitis (ARS), acute otitis media (AOM), acute uncomplicated cystitis (AC), and acute bacterial tonsillopharyngitis (ABT) before and after workshop. Total 3000 prescriptions were scored regarding rationality of the drug choice (0-10), format (0-5), instructions (0-4), legibility (0-1) and total (0-20 points). The mean number of drug(s) and total costs per prescription were calculated. Paired samples t-test was used to compare the pre- and post score means.

***Results:*** Total pre- and post-prescribing scores (0-20) were significantly different (p=0.00 for each): ANOA (13.59±0.27, 18.33±0.18), ARS (13.26 ±0.18, 15.15 ±0.17), AOM (12.58 ± 0.26, 14.66±0.27), AC (13.53±0.17, 15.76±0.20), ABT (13.54±0.24, 15.49 ±0.28). Mean number of drugs per prescription for the indications in the pre-test and post-test were: ANOA (1.24 ±0.29, 1.02±0.01, p=0.00), ARS (2.08±0.04, 2.00±0.04, p=0.16), AOM (1.66±0.04 and 1.69±0.03, p=0.54), AC (1.55±0.04, 1.39±0.03, p=0.00) and ABT (2.10±0.05, 1.81±0.05, p=0.00). Mean costs per prescription in Turkish Liras: ANOA (6.31±0.29, 4.60±0.05, p=0.00), ARS (13.80±0.38, 4.63±0.04, p=0.00), AOM (10.18±0.28, 4.41±0.07, p=0.00), AC (11.33±0.21, 10.68±0.18, p=0.01) and ABT (12.03±0.34 and 10.41±0.35, p=0.00).

***Conclusion:*** Training produced a significant improvement in rational prescribing.

## INTRODUCTION

The principles of clinical pharmacology are the basis of good patient-centered prescribing. Many would argue that teaching medical students to prescribe medicines is currently the greatest challenge in modern undergraduate education.^1^ Some studies suggest that the scope of interns’ prescribing practice is limited.^2^ The opportunities for students to ‘pre-prescribe’ under supervision, to gain experience from junior doctors or to undertake simulated prescribing exercises should be considered vital developments that might help to move beyond the current ‘in at the deep end’ experience.^1^

Training is an important intervention for rational pharmacotherapy (RP).^3^^-^^6^ In Turkey^3^^,^^7^^-,^^9^ and other countries, there are many curriculums^10^^-^^12^ and technical programs^13^ to improve the prescribing skills. We aimed to investigate the effect of our workshop for interns in rational pharmacotherapy on the rationality, cost and number of drugs prescribed in this study.

## METHODS

***Approval of ethics committee: ***Ondokuzmayis University Medical School Ethics Committee approved the Clinical Skills Project 2005- T449 and the study was a part of curriculum.

***Study design:*** One-day rational pharmacotherapy workshops have been organized for the interns during their one-month family medicine clerkship in the Department of Family Medicine at Ondokuzmayis University in Samsun, Turkey. In this study, we evaluated all prescriptions written by the interns on the first day and last day of the clerkship between July 2009 and November 2012. We excluded the prescriptions of the 40 students who did not complete their prescription task.

On the first day of the clerkship, before being introduced to RP, the participants were asked to prescribe a medication for an ANOA, ARS, AOM, AC and ABT case (Addendum 1).

***Workshop:*** The prescriptions were analyzed, and the following day, feedback was given and workshop started. A small presentation was used to correct their mistakes. The presentation was based on the WHO's Good Prescribing Guide.^14^ After reading and discussion, the correct prescription of rational drugs was taught according to the Primary Care Guidelines of the Ministry of Health.^15^ The prices of the drugs were found via updated internet resources. 

During the teaching session, the interns worked through the cases collaboratively in small groups and answered the questions posed by the medication prescribing framework. The session focuses on pharmacotherapeutic alternatives, clinical evidence. The learning experience is not only guided and structured, but also interactive and flexible, encouraging discussion and debate. Potential drug information resources were also introduced and utilized during the sessions. Pub-med and Cochrane Databases were used as additional resources. Through these steps, interns had an opportunity to identify all pharmacotherapeutic possibilities available to treat the patient, then use a rational and systematic approach to decision making that combines best practices with patient-centered care.

After completing the training in the second day, the interns continued their clerkship with the family doctors in the primary care centers and family medicine residents in the Department of Family Medicine. At the end of the clerkship, the students were asked again to prescribe for the same cases, and a prescription analysis was performed.

The authors, who have been trained in the WHO/ Groningen model of problem-based RP teaching, participated in the teaching/facilitating activities. The sessions were guided by the same family medicine teachers for all groups. The teacher’s role was to focus on the diagnostic knowledge and skills required for the case scenario, provide role modeling and coaching for the use of the framework, identify common issues from daily practice, and share real-life experiences. 

All interns who took the workshop and completed prescriptions were involved in our study. Prescriptions were evaluated and scored by another staff of the department to guarantee blinding. The participant’s therapeutic competence was also tested at the pre- and post-test phases to evaluate the effectiveness of the workshop. We approached scoring of the prescription part of the study in the same manner as Akici et al.^3^ Scores:

**A: Rationality of the Drug Choice** **(0-10 points):** Regarding efficacy (0-2.5), safety (0-2.5), suitability (0-2.5), and cost (0-2.5).

**B: Format of the Prescription** **(0-5 points):** Presence of date (0-1), physician’s name (0-1) signature (0-1), patient’s name (0-1), diagnosis (0-1). 

**C:** **Instructions** **(0-4 points): **Drug use (PO, IM etc) (0-1), dose (0-1), warnings (0-1), total amount to be delivered (0-1). 

**D:** **Legibility** **(0-1 point)**

The mean number of drug(s) and total costs per prescription were also calculated. We used the paired samples t-test to compare the pre- and post-score mean in five indications for each student. A value of p<0.05 was accepted as being statistically significant.

## RESULTS

Total 300 interns completed 3000 pre and post-prescriptions. Total pre- and post-prescribing scores (0-20) were significantly different (p=0.00) for each indication: ANOA (13.59 ± 0.27 and 18.33 ± 0.18), ARS (13.26 ± 0.18 and 15.15 ± 0.17), AOM (12.58 ± 0.26 and 14.66 ± 0.27), AC (13.53 ± 0.17 and 15.76 ± 0.20), and ABT (13.54 ± 0.24 and 15.49 ± 0.28). All data are presented (rationality, format, instructions, legibility) in Tables I-IV.

Mean number of drugs per prescription for the indications in the pre-test and in the post-test were: ANOA (1.24 ± 0.29 and 1.02 ± 0.01, p=0.00), ARS (2.08 ± 0.04 and 2.00 ± 0.04, p=0.16), AOM (1.66 ± 0.04 and 1.69 ± 0.03, p=0.54), AC (1.55 ± 0.04 and 1.39 ± 0.03, p=0.00) and ABT (2.10 ± 0.05 and 1.81 ± 0.05, p=0.00).

Mean cost of the prescriptions in Turkish Liras (TL) (1 USD=1.78 TL) for the indications in the pre-test and in the post-test were: ANOA (6.31 ± 0.29 TL and 4.60 ± 0.05 TL, p=0.00), ARS (13.80 ± 0.38 TL and 4.63 ± 0.04 TL, p=0.00), AOM (10.18 ± 0.28 TL and 4.41 ± 0.07 TL, p=0.00), AC (11.33 ± 0.21 TL and 10.68 ± 0.18 TL, p=0.01) and ABT (12.03 ± 0.34 TL and 10.41 ± 0.35 TL, p=0.00).

## DISCUSSION

Small-group teaching and learning has achieved an admirable position in medical education.^16^ The successful delivery of learning in clinical pharmacology will involve a variety of learning styles, including lectures and problem-based tutorials, but the content should be centered on inquisitive rather than passive learning.^2^^,^^17^ In our study, we used presentation, readings, discussion, and research groups in small batches.

**Table-I:** Scores Regarding Rationality of the Drug Choice*.

*Indication *	*Pre-test score (0-10)*	*Post-test score (0-10)*	*P value*
ANOA	5.05 ± 0.22	9.36 ± 0.13	0.00
ARS	4.69 ± 0.13	6.28 ± 0.15	0.00
AOM	4.68 ± 0.16	6.28 ± 0.18	0.00
AC	5.11 ± 0.13	6.98 ± 0.16	0.00
ABT	5.08 ± 0.19	6.78 ± 0.23	0.00

* N= 300 interns, 3000 prescriptions.

**Table-II:** Scores Regarding Format of the Prescription

*Indication*	*Pre-test score (0-5)*	*Post-test score (0-5)*	*P value*
ANOA	4.48 ± 0.06	4.60 ± 0.05	0.09
ARS	4.47 ± 0.06	4.61 ± 0.05	0.02
AOM	4.15 ± 0.09	4.36 ± 0.08	0.04
AC	4.45 ± 0.06	4.58 ± 0.06	0.08
ABT	4.60 ± 0.06	4.48 ± 0.06	0.76

**Table-III:** Scores Regarding Instructions

*Indication*	*Pre-test score (0-4)*	*Post-test score (0-4)*	*P value*
ANOA	3.05 ± 0.05	3.41 ± 0.04	0.00
ARS	3.09 ± 0.05	3.31 ± 0.04	0.00
AOM	2.80 ± 0.06	3.10 ± 0.06	0.00
AC	2.99 ± 0.05	3.42 ± 0.10	0.00
ABT	3.02 ± 0.05	3.27 ± 0.05	0.00

**Table-IV:** Scores Regarding Legibility

*Indication*	*Pre-test score (0-1)*	*Post-test score (0-1)*	*P value*
ANOA	0.96 ± 0.01	0.99 ± 0.01	0.05
ARS	0.97 ± 0.01	0.98 ± 0.01	0.26
AOM	0.91 ± 0.02	0.94 ± 0.01	0.14
AC	0.96 ± 0.01	0.97 ± 0.01	0.25
ABT	0.96 ± 0.01	0.96 ± 0.01	0.79

Developing a personal formulary and using an existing formulary both increase the competence of medical students in rational prescribing.^18^ In another study, it was found that interns base their prescribing on their teachers’ choices^.^^19^ After taking workshop, our students were asked to write their own prescriptions.

There are some studies that indicate the students perceive RP training to be useful.^20^^,^^21^ In our study, we evaluated the prescription scores before and after the training, which is more objective measurement than the students’ perceptions.

Students are overwhelmed by the large number of drugs. A limited formulary offers a learning target that is realistic. The list should comprise drugs that are commonly used in treating common illnesses.^1^ In our case, our interns may prepare their own formulary.

Several reports show that training workshops on RP improve the skills of the trainees.^2^^,^^4^^,^^22^^-^^24^ Postgraduate workshops were also found effective.^17^^,^^25^ The results of a study show that even a short training workshop can significantly improve the ability of students to solve written patient problems.^26^ In our study, at the pre-test, the interns exhibited a common irrational prescribing habit in that the rules of prescribing were not followed, e.g., bad handwriting, no information about the drug (e.g., after meal, before meal, how many times), etc. and it all improved after the workshop.

Several strategies for regulating prescribing practices have been proposed, such as formulary replacement, health care provider education, and feedback activities.^27^ In our study, we used all three strategies for better prescribing. Prescription analysis reveals better results for Rational Pharmacotherapy Educated (+) interns regarding the number of drugs/prescription and treatment costs^28^ similar to our study.

Use of the prescribing guidelines also had an impact on the number of drugs/prescription and treatment costs.^29^^-^^32^ The problem of prescribing irrational antibiotics was found to be related to doctors not knowing of the evidence from clinical trials.^33^ Our students reviewed the guidelines and evidence on the drugs and number of drugs per prescription decreased after the workshop.

Legibility and handwriting is important in writing a prescription.^34^^,^^35^ In our study, legibility did not improve after the training workshop. However, number of inappropriate drugs and costs decreased after the interventions^36^^-39^ Mean number of drugs also decreased in our study.

## CONCLUSION

Training produced a significant improvement in prescribing skills. However, we could not evaluate long term effects and this may be a limitation for our study. Training on rational pharmacotherapy should be considered in undergraduate medical education.

## Notes

***Note: ***The study is original and has not been published or is not currently under consideration by another journal.

## Addendum 1: Case vignettes used during teaching.

Mrs. ND; 57 years old, was diagnosed with AOA in both the knees.

Mr. MB; 22 years old, was diagnosed with ARS.

AK; four years old, was diagnosed with AOM in his right ear.

Mrs. AA; 37 years old, was diagnosed with AC.

Mrs. AB, 24 years old, was diagnosed with ABT.
